# Circulating Tumor Cells: How Far Have We Come with Mining These Seeds of Metastasis?

**DOI:** 10.3390/cancers16040816

**Published:** 2024-02-17

**Authors:** Vijay Radhakrishnan, Jussuf T. Kaifi, Kanve N. Suvilesh

**Affiliations:** 1 Department of Surgery, Ellis Fischel Cancer Center, Roy Blunt NextGen Precision Health Institute, University of Missouri, Columbia, MO 65212, USA; vrfgz@missouri.edu (V.R.); kaifij@health.missouri.edu (J.T.K.); 2 Harry S. Truman Memorial Veterans’ Hospital, Columbia, MO 65201, USA

**Keywords:** circulating tumor cells, epithelial to mesenchymal transitions, metastasis, CTC-derived models, minimal residual disease, biomarker, actionable mutations, chemotherapy, tyrosine kinase inhibitors, targeted therapy, immunotherapy, clinical trials, progression-free survival, overall survival

## Abstract

**Simple Summary:**

Circulating tumor cells are cancer cells that detach from the primary tumor and enter the bloodstream. These cancer cells in the blood stream eventually result in secondary tumor growth referred to as metastasis. Research on circulating tumor cells is crucial because they can provide valuable insights into cancer progression and treatment response that enhances the patient outcomes. Findings from circulating-tumor-cell-based research can also shed light on cancer metastasis, drug resistance, and tumor evolution, ultimately benefiting the research community by advancing our understanding of cancer biology and guiding the development of innovative treatments. In this review, we have attempted to consolidate the milestones in CTC-based research and their utility in understanding the biology of cancer from origin to progression.

**Abstract:**

Circulating tumor cells (CTCs) are cancer cells that slough off from the tumor and circulate in the peripheral blood and lymphatic system as micro metastases that eventually results in macro metastases. Through a simple blood draw, sensitive CTC detection from clinical samples has proven to be a useful tool for determining the prognosis of cancer. Recent technological developments now make it possible to detect CTCs reliably and repeatedly from a simple and straightforward blood test. Multicenter trials to assess the clinical value of CTCs have demonstrated the prognostic value of these cancer cells. Studies on CTCs have filled huge knowledge gap in understanding the process of metastasis since their identification in the late 19th century. However, these rare cancer cells have not been regularly used to tailor precision medicine and or identify novel druggable targets. In this review, we have attempted to summarize the milestones of CTC-based research from the time of identification to molecular characterization. Additionally, the need for a paradigm shift in dissecting these seeds of metastasis and the possible future avenues to improve CTC-based discoveries are also discussed.

## 1. Background

**Figure 1:**
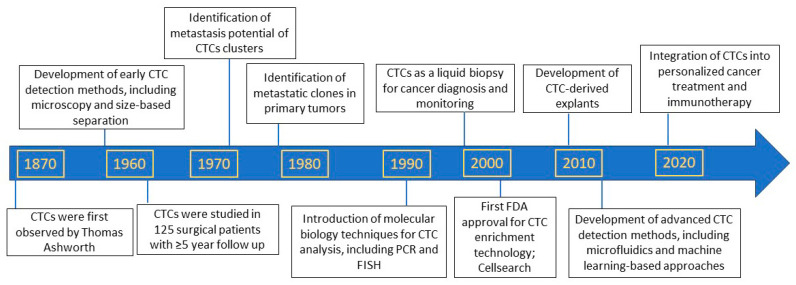
Important milestones in circulating-tumor-cell-based research [1,2,3,4,5,6,7,8,9,10,11,12,13,14,15,16,17,18].

Preliminary observations of circulating tumor cells (CTCs) date back to the early 19th century, and Thomas Ashworth is credited for proposing their potential role in cancer metastasis. Ashworth observed that cancer patients’ blood contained tumor cells and suggested that these cells could migrate through the bloodstream to establish secondary tumors in distant organs. This observation formed the basis for the subsequent identification and characterization of circulating tumor cells (CTCs) as ‘micrometastatic seeds’ or ‘seeds of metastasis’. No significant breakthroughs were made for approximately a century following the initial discovery of circulating cancer cells. Nonetheless, the research community began paying attention to these circulating cancer cells in the late 1950s, and since then, CTC-based studies have undergone a fascinating evolution that continues to the present day (Figure 1). We now know that single CTCs can be sequenced, and large next-generation sequencing (NGS) data from multi-center studies can be generated to explore the process and progression of cancer from locoregional disease to widespread metastasis. The basic understanding is that CTCs are extravasated from the primary tumor into the bloodstream through a complex process, where they act as surrogates reflecting the characteristics of the tumor itself. Our review aims to elucidate the chronology of CTC-based discoveries and their significance in enhancing cancer management and patient prognosis. The rarity of CTCs within the vasculature engendered uncertainty regarding their potential in mediating metastasis; however, it is now well established that even a single CTC possesses tumorigenic capacity and can serve as a metastatic source.

## 2. Discovery of Circulating Tumor Cells as Precursors of Metastasis

The discovery of CTCs as precursors of metastasis has been a transformative journey spanning over a century. In 1889, Stephen Paget first proposed the “seed and soil” hypothesis, suggesting that cancer cells travel through the bloodstream and grow in distant organs [19]. The fundamental idea of the “seed and soil’ hypothesis was a metaphor comparing cancer cell migration to plant germination. Similar to the fact that seeds dispersed by a plant can only thrive in congenial soil, cancer cells traveling through the vasculature require a receptive environment to germinate and grow in distant organs. Although the hypothesis sufficiently illustrated the concept of distant growth, it initially lacked consideration [20]. It was not until the mid-20th century that observational and experimental evidence emerged, demonstrating that cancer cells from primary tumors could spread to secondary sites through the circulation. Ashworth’s initial observation of epithelial cells in the blood of a dying cancer patient in 1869 suggested the basis for primary tumor-derived circulating cells [1]. Following Ashworth’s observation, various studies reported tumor cells in the postmortem blood of patients with different cancers. A few studies also reported tumor cells in the blood collected from patients a few hours prior to death [2]. The first study on CTCs in 125 surgical patients having different cancers with a long follow up of 5 years was evidenced in the year 1959 [3]. Later, similar studies were conducted by several investigators to observe the dissemination of CTCs during and after operative procedures [4,5]. Fidler and Kripke experimentally demonstrated that metastasis results from pre-existing variant cells within a malignant tumor. They showed that clones derived in vitro from a parent culture of murine malignant melanoma cells varied greatly in their ability to produce metastatic colonies in the lungs upon intravenous inoculation into syngeneic mice. This study was a breakthrough in demonstrating the heterogenous nature of primary tumor and clonal selection during metastasis [6]. Moving forward, the limited number of CTCs in the patients’ blood stalled the studies to demonstrate their metastasis initiating ability or tumorigenic potential. Firstly, and most importantly, the extensive availability of cells is imperative for their functional and biological characterization. The scarcity of CTCs in the bloodstream historically presented both opportunities and challenges for researchers. Scientific community shifted their focus on enrichment of CTCs and to date different antibody-based positive and negative enrichment technologies [7] and microfluidics-based isolation methods (Table 1) are available. Further milestones uncovering the metastasis initiating potential of CTCs are evidenced due to the availability of better CTC enrichment technologies. The growth of xenograft tumors upon implantation of CTCs enriched from the peripheral blood of human patients, coupled with the association of high CTC numbers in circulation with disease recurrence in patients, provided conclusive evidence that CTCs are indeed the seeds of metastasis [8,21,22,23,24,25,26,27]. Further, to assess the utility of CTCs in forecasting patient outcomes and evaluating their predictive value as biomarkers, numerous studies have been conducted that involve detecting and enumerating CTCs in cancer patients, followed by tracking their clinical progress [9,10]. This has enabled researchers to explore the relationship between CTCs and patient prognosis, and to determine the accuracy of CTCs as indicators of disease progression.

## 3. Circulating Tumor Cells as Biomarkers to Predict Patient Prognosis

Development of the CellSearch system in the early 2000s and the U.S. Food and Drug Administration approval in 2004 enabled the detection, enumeration, and characterization of CTCs in numerous clinical trials involving breast, prostate, and colorectal cancer patients [11,41]. Since then, numerous studies have consistently shown that CTCs are associated with poor prognosis and can predict disease recurrence. This technology advancement elevated liquid biopsy in cancer research wherein CTCs from the peripheral blood of patients with different cancer types were analyzed to predict the prognostic effect. For example, a seminal study in breast cancer patients found that high CTC counts were linked to decreased overall survival [42]. Similarly, in colorectal cancer, high CTC counts have been linked to worse overall survival [43,44]. Clinical utility of CTCs as disease prognosticators were shown in both castration resistant and sensitive prostate cancer patients [45]. Likewise, positive CTC counts were shown to be common in advance gastric cancer patients who presented with diffused histologic tumor types and distant metastases. Further, the study also showed that progression-free survival of CTC-positive patients was significantly shorter than that of CTC-negative patients indicating the independent prognostic potential of CTCs in gastric cancer [46]. The major drawback of the Cellsearch system is the enrichment of CTCs based on the expression of epithelial cellular adhesion molecule (EpCAM) [23]. In certain cancers like non-small-cell lung cancer (NSCLC), EpCAM expression will be low due to epithelial to mesenchymal transition, and the Cellsearch system may not be the suitable platform to enrich and enumerate CTCs. Owing to the limitations of the Cellsearch system, the detection and analysis of CTCs have become more sophisticated with advancements in non-epitope-dependent technologies like microfluidics and filtration methods. Several groups including ours utilized non-epitope-dependent technologies to study the biomarker potential of CTCs in predicting prognosis of cancer patients. Using a size-based microfiltration method, one study demonstrated the prognosis of head and neck squamous cell carcinoma patients in correlation to CTC numbers [47]. Similarly, the prognostic value of CTCs was demonstrated in NSCLC patients and screening subjects [48,49,50]. Additionally, C-X-C chemokine receptor type 4 (CXCR4) expression on circulating pan-cytokeratin-positive cells was shown to be associated with survival in patients with advanced non-small-cell lung cancer [51]. Another major advantage of non-epitope-dependent technologies is the identification of multi-phenotypic subtypes of CTCs and CTC clusters or microemboli [48,49]. For instance, CTC clusters are shown to be more aggressive phenotypes than individual CTCs [52]. CTC clusters are defined as two or more group of tumor cells with an intact nucleus [25]. Although less abundant in circulation, CTC clusters possess higher metastatic potential and elevated expression of epithelial-to-mesenchymal transition markers and stemness genes compared to individual CTCs [11,53,54,55]. There are two types of CTC clusters: homotypic and heterotypic. Homotypic clustering occurs through adhesion molecule interactions (e.g., CD44, cadherin, desmoglein, ICAM1, and desmocollin), which stabilizes clusters and activates downstream pathways that enhance invasiveness and migration [54,56,57,58]. Patients with elevated individual CTC counts exhibit significantly poorer overall survival, and this association is further exacerbated in patients with higher CTC cluster levels [48,59,60]. On the other hand, CTC heterotypic clusters are characterized as tumor cells forming clusters with other cell types such as leukocytes, neutrophils, fibroblasts, platelets, and myeloid derived suppressor cells [61,62,63,64,65]. Patients with CTC–leukocyte clusters have shown worse overall survival compared to patients without CTC–leukocyte clusters [66]. CTCs clustered with neutrophils and fibroblasts have been shown to have higher cell division and invasion and migration, respectively [61,62]. Platelets clustered with CTCs are believed to aid them in escaping from immune surveillance [67]. Furthermore, analysis of CTCs has also revealed insights into tumor biology, such as the presence of putative cancer stem cells and circulating tumor microemboli [68]. Overall, the use of CTCs as biomarkers has the potential to revolutionize personalized cancer care, and ongoing research is exploring new frontiers in CTC analysis, including their potential use as liquid biopsies and predictors of cancer relapse.

## 4. Circulating Tumor Cells as Biomarkers to Predict Anti-Cancer Therapy Responses

**Figure 2:**
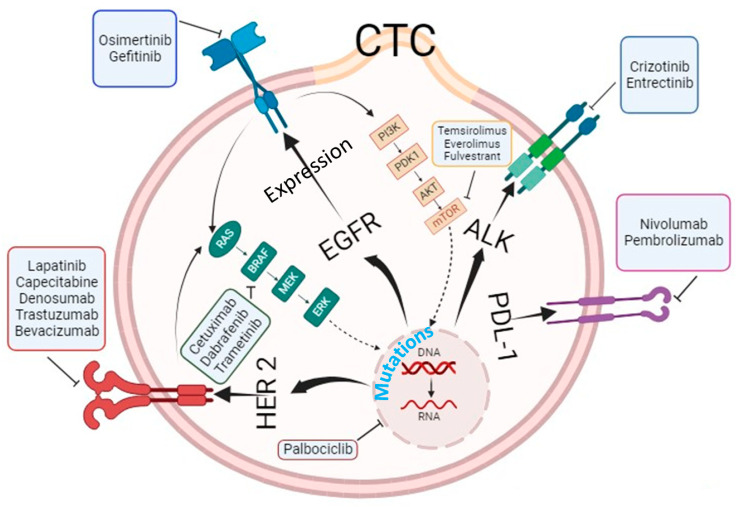
Anti-cancer treatment strategies based on CTC expression and mutational analysis. Created using Biorender (https://biorender.com accessed on 3 January 2024).

CTCs have potential uses beyond cancer diagnosis and prognosis. CTCs may serve as a means to monitor cancer minimal residual disease after treatment. CTCs offer a non-invasive way to assess cancer progression and treatment response, potentially reducing the need for invasive biopsies. As outlined below, a variety of neoadjuvant or adjuvant anti-cancer therapies have been tailored based on the expression and mutational analysis performed in CTCs (Figure 2). Even the response to tailored treatment strategy has been shown to corelate with CTCs at baseline, during therapy and post-therapy.

### 4.1. Circulating Tumor Cells as Biomarkers to Predict Chemotherapy Responses

Research has shown that CTCs can be used to predict chemotherapy response in various cancer types [45,69,70]. For instance, a study found that the presence of CTCs in breast cancer patients before systemic adjuvant treatment and after completion of chemotherapy was associated with poor prognosis and reduced overall survival [71]. In metastatic castration-resistant prostate cancer patients receiving first-line docetaxel-based therapy, fewer than 5 CTCs per 7.5 mL of pre-therapy was associated with median overall survival of 26 months and that with 5 or more CTCs per 7.5 mL had a survival of 13 months. Increasing CTC counts at three weeks were associated with considerably lower overall survival, suggesting that the baseline CTC count is a reliable, independent biomarker to determine therapy outcome [70]. In lung cancer, CTCs isolated before chemotherapy were shown to predict treatment response and disease recurrence [72]. In patients with locally advanced rectal cancer, standard treatment consists of neoadjuvant chemoradiation followed by total mesorectal excision [73]. In the context of rectal cancer treatment, 5-fluoro uracil is a commonly employed cytotoxic agent that targets the enzyme thymidylate synthase [74]. On the other hand, RAD23 homolog B is a protein that plays a role in the nucleotide excision repair process and is inducible by genetic damage triggered by radiation therapy [75]. One study investigated CTCs in 30 locally advanced rectal cancer patients before treatment and found that CTC counts decreased after chemoradiation in patients who exhibited pathological complete or partial response [75]. Notably, thymidylate synthase expression in CTCs was absent in patients with complete response, but present in 83% of non-responders. In contrast, RAD23 homolog B expression was observed in all non-responders, highlighting the value of combining molecular analysis of CTCs with enumeration to predict treatment outcomes accurately. Additionally, the inclusion of expression analysis of thymidylate synthase and RAD23 homolog B in CTCs increased the sensitivity of the biomarker analysis in predicting treatment outcome [75]. Similarly, combining CTC enumeration and evaluation of serological cell death biomarkers has been demonstrated to be a valuable strategy for predicting chemotherapy outcome in small-cell lung cancer patients. In another study, blood samples from small-cell lung cancer patients receiving chemotherapy showed a decrease in CTC numbers following treatment, reflecting treatment response. Conversely, serological cell death biomarkers, specifically M30 and nucleosomal DNA, exhibited elevated levels at 48 h post-treatment, indicating early response and severe toxicity [76]. Furthermore, the persistent presence of CTCs after chemotherapy in colon cancer patients has been shown to be strongly correlated with reduced disease-free survival and overall survival. Additionally, analysis of CTCs post-chemotherapy has been found to be more accurate in detecting relapse compared to analysis of the well-established biomarker carcinoembryonic antigen, highlighting the potential of CTC analysis as a valuable tool for predicting treatment outcomes in advanced colon cancer patients undergoing chemotherapy [77]. Pancreatic ductal adenocarcinoma, a highly aggressive cancer, necessitates effective biomarkers to monitor disease progression in chemotherapy-treated patients. A study demonstrated that high CTC numbers pre- and post-chemotherapy are a prognostic factor for poor overall survival and progression-free survival in advanced pancreatic ductal adenocarcinoma patients. Notably, CTCs with high activated leukocyte cell adhesion molecule (ALCAM) and POU class 5 homeobox 1B (POU51B) expression correlated with shorter survival times. The study also revealed two distinct gene expression profiles in CTCs before and after chemotherapy; the epithelial genes (EpCAM, VEGFA) were dominant before chemotherapy and the stemness/pluripotency genes (ALCAM, POU51B) were enriched after chemotherapy, suggesting dynamic changes in CTC biology [78].

The above-mentioned studies clearly demonstrate that the analysis of CTCs can help identify specific biomarkers associated with chemotherapy resistance and efficiently aid in disease monitoring. The presence of CTCs with certain genetic mutations or expression of specific proteins can predict poor response to chemotherapy [79]. Furthermore, CTCs can be used to monitor treatment response in real-time, allowing for adaptive therapy strategies [80].

### 4.2. Circulating Tumor Cells as Biomarkers to Predict Targeted Therapy Responses

Targeted therapy is a type of cancer treatment that targets specific molecules involved in cancer growth and progression [81]. For example, a targeted therapy can be receptor tyrosine kinase inhibitor or monoclonal antibody targeting a specific molecule. These therapies can be more effective and less toxic than traditional chemotherapy. Cancer treatment has made significant strides with the development of targeted therapies, designed to attack specific cancer-driving proteins or pathways. However, resistance to targeted therapy can develop through various mechanisms, including genetic mutations and epigenetic alterations [81,82]. Recent studies have investigated the association between CTCs and targeted therapy in cancer patients and shown that besides predicting chemotherapy responses, CTCs are also useful in monitoring targeted therapy. Studies have shown that CTCs can predict responses to targeted therapies, such as tyrosine kinase inhibitors (TKIs), in lung cancer patients. For example, one study attempted to investigate CTCs with epidermal growth factor receptor (EGFR) mutations before and after TKI treatment and found that an increase in the number of cells was associated with tumor progression, with the emergence of additional EGFR mutations in some cases [83]. CTCs from breast cancer patients exhibited increased expression of the phosphatidylinositol-4,5-bisphosphate 3-kinase catalytic subunit alpha (PI3KCA) hotspot mutation during treatment with the CDK4/6 inhibitor palbociclib, suggesting the emergence of resistance [84]. CTCs from breast cancer patients with human epidermal growth factor receptor (HER2)-positive tumors exhibit increased expression of HER2, which can help predict the response to HER2-targeted therapies like trastuzumab (Herceptin) [85]. Similarly, CTCs from lung cancer patients with EGFR mutations can predict the response to EGFR-targeted TKIs like gefitinib (Iressa) [86]. Likewise, CTCs were enumerated in RAS-BRAF wild-type colorectal cancer patients receiving third-line anti-EGFR monoclonal antibodies, cetuximab or panitumumab and found that CTC status assessed early on during targeted therapy may predict treatment failure in advance compared to imaging-based tools [87]. Genetic and phenotypic profiling is critical in selecting the suitable targeted therapy because often genetic and phenotypic characteristics of CTCs differs from that of the primary tumor [88]. CTCs and primary tumor cell phenotypes are not always identical, and CTC phenotypes can be transient. For example, inconsistent HER2 expression was observed between the primary tumor and CTCs of gastric cancer patients. HER2-positive CTCs were found in the blood of 17 patients with 54 HER2-negative primary tumors. Similarly, the blood of five patients with HER2-positive primary tumors produced HER2-negative CTCs, suggesting the importance of phenotypic or genetic characterization of CTCs to tailor precision medicine [89]. Additionally, CTCs were demonstrated to act as biomarkers for monitoring responsiveness to BRAF-targeted therapy in advanced melanoma patients [90]. Specifically, BRAF-mutated CTCs were associated with treatment response to BRAF inhibitors. Analysis of blood samples from advanced BRAF-mutated melanoma patients revealed a decrease in CTC numbers with treatment, accompanied by a positive correlation between CTC numbers and tumor regression. Notably, one patient with a BRAF V600E mutation in the primary tumor but not in the lymph nodes highlighted the heterogeneity of the BRAF genotype between the primary tumor, metastasis, and CTCs. Furthermore, this patient’s blood contained a variety of BRAF-mutated CTCs, including V600R, V600M, V600A, K601E, K601R, and A598V, in addition to V600E. While most BRAF-mutated CTCs disappeared during treatment, BRAF A598V and wild-type CTCs persisted even after other BRAF-mutated CTCs were cleared [90]. These findings suggest that CTC analysis could guide targeted therapy selection and monitoring.

### 4.3. Circulating Tumor Cells as Biomarkers to Predict Immunotherapy Responses

Immunotherapy has revolutionized cancer treatment, offering a promising approach to harness the body’s immune system to combat cancer [91]. However, predicting which patients will respond to immunotherapy remains a significant challenge [92]. Another exciting area of research involves using CTCs to predict response to immunotherapies. Recently accumulated evidence suggested that CTCs can be used as biomarkers to predict response to immunotherapy in various cancer types. The assessment of programmed death-ligand 1 (PD-L1) expression is a clinical practice for selecting patients for immune checkpoint inhibitor therapy or immunotherapy, wherein PD-L1 serves as a biomarker [93]. This approach is based on the understanding that PD-L1 expression on tumor cells can predict the likelihood of a response to immunotherapy, and its evaluation has become a crucial step in personalizing cancer treatment. Studies have shown that CTCs can be analyzed for their expression of immune checkpoint proteins like PD-L1, which can help predict response to immune-targeting drugs like checkpoint inhibitors. For instance, PD-L1 expression of CTCs from 127 NSCLC patients clearly demonstrated their utility in predicting immunotherapy response. Major findings of the study showed an increase in PD-L1-positive CTCs in all patients with disease progression, while no change or a decrease in PD-L1-positive CTCs was observed in responding patients [94]. In another study of 24 advanced-stage NSCLC patients treated with Nivolumab, CTCs were analyzed for PD-L1 expression at baseline, 3, and 6 months post therapy initiation [95]. A correlation between PD-L1-expressing CTCs and outcome was observed. Specifically, at baseline and 3 months, the presence of CTCs and PD-L1 expression on their surface were associated with poor patient outcomes. On the other hand, at 6 months, patients with PD-L1-negative CTCs achieved clinical benefit, while patients with PD-L1-positive CTCs experienced progressive disease [95]. In the context of head and neck cancers, anti-PD1 agents have become the standard of care for chemotherapy refractory, recurrent or metastatic head and neck squamous cell carcinoma patients [96]. A prospective study involving 113 locally advanced head and neck squamous cell carcinoma patients investigated the correlation between PD-L1 expression in CTCs at baseline, after two cycles of chemotherapy, and at the end of concurrent chemoradiotherapy with progression-free survival and overall survival. Specifically, patients with CTCs that overexpressed PD-L1 at the end of treatment exhibited shorter progression-free survival and overall survival. Conversely, the absence of PD-L1 overexpression at the end of treatment was strongly associated with complete response [97]. This study underscores the importance of adjuvant PD1 inhibitors in HNSCC patients in whom PD-L1-positive CTCs are detected at the end of curative treatment. Furthermore, an analysis of blood samples from 25 patients with muscle-invasive and metastatic bladder cancer revealed that individuals with a high burden of PD-L1-positive and CD45-negative CTCs and a low burden of apoptotic CTCs exhibited poorer overall survival. This finding suggests that the combination of PD-L1 expression and low apoptotic activity in CTCs may serve as a prognostic marker and potential guide for clinicians about patients’ suitability for immunotherapy [98]. These findings, including other studies, suggest that CTCs can be used to monitor changes in the tumor microenvironment during immunotherapy treatment, allowing for real-time assessment of treatment effectiveness [99,100]. Overall, the analysis of CTCs offers a promising avenue for non-invasive biomarker development, with potential applications in predicting treatment response and detecting cancer recurrence. As research continues to advance, the clinical utility of CTCs is likely to expand, providing valuable insights into cancer biology and improving patient outcomes.

## 5. Molecular and Genetic Characterization of Circulating Tumor Cells beyond Enumeration to Identify Actionable Mutations

The analysis of CTCs has evolved beyond mere enumeration, with advances in technologies enabling molecular and genetic characterization. This allows for the detection of specific genetic mutations, the expression of surface proteins, and analysis of gene transcripts in CTCs. Molecular characterization of CTCs using different techniques (Table 2) including array comparative genomic hybridization, reverse transcription-polymerase chain reaction (RT-PCR), fluorescence in situ hybridization (FISH), and NGS has been accomplished [12,101,102,103]. These studies suggest that CTC genetic analysis may be more appropriate than fresh tissue biopsy for studying tumor heterogeneity and clonal evolution. Single-cell RNA sequencing revealed the heterogeneity of CTCs in breast cancer, identifying distinct subpopulations with different gene expression profiles [104]. Our group identified the distinct single cell heterogeneity between circulating-tumor-cell-derived xenografts and patient primary-tumor-derived xenografts of NSCLC by single nuclear RNA sequencing [105]. Digital PCR has proven to be a valuable technique to detect EGFR and Kirsten rat sarcoma virus (KRAS) gene mutations in CTCs from non-small-cell lung cancer patients, demonstrating its potential as a liquid biopsy for monitoring treatment response [106,107]. Additionally, researchers have used molecular and genetic characterization of CTCs to investigate tumor evolution and resistance to therapy. Whole-exome sequencing of CTCs identified genomic alterations driving resistance to androgen receptor-targeted therapy in prostate cancer [108]. Further, expression analysis and NGS of CTCs have been shown to be useful tools to assess intra patient or intra tumoral heterogeneity. EGFR gene amplification and heterogenous expression was observed between CTCs from the same patient with colorectal cancer. Additionally, study also showed that KRAS and PIK3CA mutations were detected in only 5 out of 15 CTCs and 14 out of 36 CTCs, respectively, from the same patients [109]. Utilizing CTCs to study the intra patient or intra tumor heterogeneity provides important information for the disease prognosis, drug responsiveness, and personalized treatment of cancer patients. Thanks to cutting-edge technologies, we can now analyze CTCs at the genetic, transcriptomic, and proteomic levels, which has helped bridge the knowledge gap in understanding the metastasis process and tailor precision medicine.

Numerous studies have leveraged CTCs to uncover novel actionable mutations in various cancer types in pre-treatment or during treatment. For instance, a study used CTCs to identify PIK3CA mutations in estrogen-receptor-positive breast cancer, suggesting potential benefit from PI3K inhibitors [121]. Another study identified EGFR mutations in NSCLC CTCs, which could guide targeted therapy with EGFR inhibitors [122]. Furthermore, CTC analysis has revealed novel mutations in rare cancer subtypes. A study identified BRAF V600E and V600K mutations in circulating tumor cells from patients with melanoma, indicating potential benefit from BRAF inhibitors [123]. Additionally, CTCs have also been utilized to detect gene rearrangements such as ALK, ROS1-, RET-rearrangements in NSCLC and ERG-rearrangements in prostate cancer [124]. Moreover, CTC analysis has enabled the detection of resistance mutations in real-time. CTCs collected from NSCLC patients with EGFR mutations who had received tyrosine kinase inhibitors harbored T790M mutations, suggesting potential benefit from next-generation EGFR inhibitors [83]. These findings highlight the potential of CTCs for precision oncology and warrant further investigation.

## 6. Circulating Tumor Cells as Models to Identify Metastasis Competent Signatures

CTCs are regarded as the seeds of metastasis and given their molecular and genetic profiles often differ from those of the primary tumor, it is scientifically important to investigate the mechanisms of metastasis within these cellular population to block the progression of the disease. CTCs are proven valuable tools to identify phenotypic, genetic, and epigenetic signatures associated with metastasis. CTCs from breast cancer patients with a gene expression pattern predicted metastasis to the bone, lung, and liver [8]. In a xenograft assay, the authors demonstrated that CTCs positive for EpCAM, EPCAM, CD44, CD47, and MET exhibit metastasis-initiating potential, expanding the scope beyond EpCAM+ CTCs alone. Additionally, CTCs with similar molecular signatures in patient cohorts showed a correlation with poor overall survival, supporting the findings observed in patient-derived xenografts [8]. Similarly, CTCs from breast cancer patients are found to express a signature required for brain tropism [125]. Notably, CTCs that were negative for EpCAM but positive for HER2, EGFR, HPSE, and Notch1 were found to be highly invasive and capable of generating brain and lung metastases when xenografted in nude mice, highlighting the brain competent metastasis signature and limitations of the CellSearch system in capturing these breast cancer circulating cells [125]. Likewise, CTCs from colorectal cancer patients were shown to acquire a few features of epithelial to mesenchymal transition and increase in the expression of mesenchymal to epithelial transition, indicating a metastasis-competent phenotype [126]. In particular, all nine CTC lines generated by serial blood draw from a metastatic colorectal cancer patient exhibited expression of the oncogenes MYC and ezrin, while lacking expression of the epithelial-to-mesenchymal transition inducer gene SIX1. Conversely, the mesenchymal-to-epithelial transition activator GRHL2 and its targets were strongly expressed in all CTC lines, supporting a role in metastasis formation [126]. Furthermore, analysis of CTCs has revealed alterations in key signaling pathways that promote metastasis. For instance, a study showed that CTCs from melanoma patients needed to activate PI3K/Akt/mTOR pathway during the earliest steps of brain colonization, which enhances cell survival and migration [127]. Moreover, CTC analysis has also revealed the presence of putative cancer stem cells, which are thought to be responsible for the initiation and progression of metastasis. A study identified a subpopulation of CTCs in breast cancer patients that expressed stem cell markers and could be the reason for enhanced tumor-initiating capacity [128]. In a cohort of 38 breast cancer patients, presence of stemness and epithelial-to-mesenchymal transition in CTCs were identified by assessing the expression of stem cell markers (CD44, ALDH1, and CD133) and the epithelial-to-mesenchymal transition marker N-cadherin. Notably, all N-cadherin-positive CTCs exhibited stem features, as evidenced by co-expression of CD133 and ALDH1, whereas N-cadherin-negative CTCs lacked stem cell markers (ALDH1, CD44, and CD133), indicating a non-stem cell phenotype [128]. These findings suggest that CTCs can be a valuable tool for identifying molecular signatures and pathways associated with metastasis, which may inform the development of new therapeutic strategies to prevent or treat metastatic disease.

## 7. Real World Evidence by Circulating-Tumor-Cell-Based Clinical Trials

CTCs have emerged as a promising biomarker for cancer diagnosis and monitoring. Several clinical trials have investigated the clinical utility of CTCs in various cancer types to accumulate real world data or evidence (Table 3). CTC in patients have been monitored to determine the first, second and/or late line chemotherapy treatment outcomes in NSCLC, breast, gastric, colorectal, and pancreatic cancers [129,130,131,132,133]. Clinical trials in advanced breast cancer patients laid the foundation for scientific arguments regarding the utility of CTCs as a biomarker for treatment response. Specifically, one study found that switching cytotoxic therapy in response to an increase in CTCs during first-line therapy did not significantly impact patient survival outcomes [130]. In contrast, another study observed a correlation between early changes in CTC count and treatment response to third-line chemotherapy, suggesting potential clinical utility for CTC monitoring in this context [134]. These differences in clinical observations could be due to disparities in sample processing methods, including blood collection and storage protocols, as well as differences in patient populations and sample sizes, leading to heterogeneity in CTC characteristics. Additionally, the use of diverse analytical platforms to analyze CTCs can introduce technical variability. To address these inconsistencies, a standardized approach to defining CTCs and establishing appropriate cut-off values for each cancer type is essential. Nonetheless, CTC number was found to be significantly correlated to prognosis in gastric cancer patients treated with fluorouracil-based chemotherapy and NSCLC patients treated with standard of care chemotherapy [129,131]. Additionally, differential gene expression pattern was observed in colon cancer CTCs of the same patient during first- and second-line chemotherapy treatments, highlighting the importance of acquired biological changes in these seeds of metastasis due to selective treatment pressure [132]. Furthermore, chemosensitivity assay profiling of CTCs has been demonstrated to be a valuable tool for guiding therapy in advanced pancreatic adenocarcinoma [133].

Clinical trials to understand the clinical utility of CTCs in tailoring or monitoring targeted therapy have shown some promising evidence. For instance, it is well known that somatostatin receptor expression is highly relevant in neuroendocrine tumors. Clinical trials have demonstrated the usefulness of somatostatin receptor expression detection on CTCs for tailoring somatostatin-receptor-targeted therapies [145]. Likewise, CXCR4 expression has been studied in various cancer types including breast, lung, kidney, colon, ovarian, and brain tumors, and its overexpression is believed to be associated with invasion and migration. In an exploratory analysis of phase II study, efficacy of selective CXCR4 antagonist small cyclic peptide LY2510924 plus carboplatin-etoposide treatment was correlated with baseline CTC counts in predicting survival in small-cell lung cancer patients [148]. Additionally, CTC phenotypic changes have been investigated in breast cancer irrespective of HER2-positive or -negative status; it was found that CTCs are heterogenous compared to primary tumors and phenotyping CTCs are critical in stratifying patients for HER2-targeted therapy [168]. Similar to measuring treatment response by assessing apoptosis and necrosis in treated tumors, apoptotic CTCs were found to be associated with longer progression-free survival in ovarian cancer patients treated with mTOR-targeted inhibitor [135]. These findings suggest that CTC-based clinical trials may lead to improved cancer diagnosis and treatment strategies and CTC-based biomarkers may help optimize cancer treatment and improve patient outcomes. However, the majority of clinical trials have been conducted with advanced-stage cancer patients (Table 3) which marks the need for trials in early-stage cancer patients to prolong their survival with better treatment choices based on liquid biopsy.

## 8. Challenges and Opportunities

Despite the promise of CTCs as a liquid biopsy for cancer diagnosis and monitoring, several challenges need to be addressed. One major challenge is the rarity of CTCs in peripheral blood, which can make their detection and analysis difficult. Additionally, the heterogeneity of CTCs and other factors in the blood can lead to false negatives or false positives. Moreover, standardization of CTC isolation and analysis methods is still a work in progress, which can hinder comparison across studies. Although NGS has undergone significant advancements, it still remains a challenge to characterize CTCs due to the limited quantity of genetic material that can be obtained from these rare cells.

However, these challenges also present opportunities for innovation and advancement. For instance, development of more sensitive and specific CTC detection methods, such as machine-learning-based algorithms, can improve accuracy. Additionally, single-cell analysis of CTCs can reveal novel insights into tumor heterogeneity and evolution. Furthermore, the integration of CTC analysis with other liquid biopsy markers, like circulating DNA or RNA, can enhance diagnostic and prognostic power. Finally, investigations into the biology of CTCs can unveil new targets for cancer therapy and enable personalized treatment strategies.

Another opportunity lies in the development of CTC-based liquid biopsies for early cancer detection and screening. If CTCs can be detected and characterized in individuals without symptoms, it may be possible to identify cancer at an early stage. Additionally, CTC analysis may help identify patients at risk of cancer relapse, allowing for earlier intervention and potentially improving outcomes.

Moreover, advancements in single-cell analysis enable the study of CTCs at the single-cell level, allowing researchers to explore the genetic and epigenetic landscape of individual CTCs. This knowledge can aid in the development of targeted therapies and immunotherapies. Furthermore, researchers can leverage CTCs as a platform for drug testing and development, potentially leading to more effective and personalized treatments. Overall, while challenges persist in CTC research, ongoing advancements and collaborative efforts can overcome these hurdles, paving the way for CTCs to become a valuable tool in cancer care.

## 9. Conclusions

CTCs have emerged as a promising biomarker for cancer diagnosis, prognosis, and treatment monitoring, and research on CTCs has made significant progress in recent years, shedding light on their role in cancer metastasis. Over the past decade, substantial development has been made in CTC research, including advancements in detection and characterization techniques, identification of CTC subpopulations, and integration of CTC analysis into clinical trials. These advancements have paved the way for CTCs to become a valuable tool in cancer diagnosis and management. Evaluation of CTCs in various cancer types and investigation of their potential in combination with other liquid biopsy markers, such as circulating tumor DNA, will likely yield valuable insights into cancer biology and treatment response. However, challenges remain, including standardization of CTC enumeration and molecular characterization, further validation of CTC-based biomarkers, and exploration of CTCs in large cohorts of early-stage cancer patients. Nonetheless, the field of CTC research has made substantial strides towards unlocking the potential of these “seeds of metastasis” to improve cancer patient outcomes. As research continues to unravel the complexity of CTC biology, standard methodology for CTC enumeration including cut-off for a particular cancer type will likely be developed for widespread clinical adoption. Furthermore, the development of novel molecular technologies like NGS and analytical methods have aided in-depth analysis of CTCs and the process of metastasis. For instance, single-cell analysis and artificial intelligence-driven approaches may reveal new aspects of CTC heterogeneity and tumor evolution. Additionally, the integration of CTC analysis with other omics technologies, such as proteomics and metabolomics, could provide a more comprehensive understanding of cancer biology. Furthermore, phenotypic and molecular characterization of CTCs have clearly demonstrated the intra tumor and intra patient heterogeneity and the need for regular change in the treatment strategy leading to actual precision medicine. 

## Figures and Tables

**Table 1:** A few microfluidics-based CTC enrichment techniques.

Name	Enrichment Technique	Type (Physical or Biological)	Key Findings
Herringbone (HB)-Chip	Surface affinity	Biological	CTCs were detected in 93% of patients with metastatic disease [13].
Nano Velcro	Cell affinity	Biological	Capable of detecting, isolating, and purifying CTCs from blood samples with high efficiency for subsequent molecular analyses [28,29].
Nanoparticle-herringbone microfluidic chip (NP-HBCTC-Chip)	Surface affinity	Biological	Enhanced capture efficiency and recovery of isolated CTCs [30].
PEDOT Nano Velcro Chips	Cell affinity	Biological	Ability to achieve high cell purity as well as preserve the integrity of RNA transcripts from the purified cells [31].
CaTCh FISH	Magnetic separation/fluorescence in situ hybridization	Physical	Capture CTCs for in situ RNA analysis [32,33].
Two-stage microfluidic chip	Size and asymmetry based capturing	Physical	High rate (99%) CTC clusters recovery with 87% viability [34,35].
Bait-trap chip	In situ rolling circle amplification (RCA) method	Physical	Accurate and ultrasensitive capture of live CTCs from peripheral blood [35].
3D Palladium Filter	Lithography plus electroforming process	Physical	Enumeration and isolation of CTCs for genetic analysis [36].
Pillar-X	Bimodular microfluidic device	Biophysical	Efficiently captures both single cells and clusters and sorts them based on size, cohesiveness, and epithelial identity [37].
Dielectrophoretic field-flow-fractionation (DEP-FFF)	Batch-mode microfluidic di-electrophoresis method	Physical	70–75% capture efficiency [38,39].
Parsortix™ Cell Separation System	Microfluidic particle separation technology	Biophysical	High capture efficiency and viable CTCs for downstream analyses [40].

**Table 2:** Technologies for genetic and molecular characterization of CTCs.

Name	Advantages	Limitations	References
RT-PCR	High sensitivity for genes expressed at low levels. Less experimental time. Cost-effective.	Number of transcripts are limited. Requires pre-amplification of specific cDNA.	[14,110]
RNA in situ hybridization	High sensitivity for genes expressed at low levels. Comprehensive profiling. Less experimental time. Cost-effective.	Limited to transcripts that are used in probe designing.	[15,111]
Single-cell RNA sequencing	Complete profiling. Allows for the discovery of new annotated transcripts.	Expensive. Amplification bias during sequencing.	[16,25]
Fluorescence In Situ Hybridization (FISH)	Less experimental time. Less cost. Allows spatial information.	Limited number of genes.	[17,112]
Integrated immunostaining fluorescence in situ hybridization (iFISH)	Size-based identification of CTCs with karyotyping. Identification of epithelial or mesenchymal CTC type with genetic changes.	Limited number of genes.	[113]
Targeted DNA sequencing	High sensitivity. Cost-effective.	Limited number of genes.	[12,114]
Single-cell exome/genome sequencing	Complete profiling of exons.	Difficult to obtain whole exome/genome. False-positivity due to library amplification. Non-uniform coverage.	[18,115,116]
Bulk mass spectroscopy	Comprehensive profiling.	Limited number of proteins. Low sensitivity and low abundance.	[117,118]
Single-cell mass spectroscopy	Comprehensive profiling.	Not widely used. Not well established.	[119,120]

**Table 3:** CTC-based clinical trials.

Trial Number, Year of Completion, Study Type and Phase.	Name	Cancer Type	Cancer Stage and Other Information	Key Findings
NCT00429793. 2012. Interventional. Phase 2.	NA	Ovarian Cancer	Advanced. Grade 1,2,3. Tumor types-adenocarcinoma, clear cell carcinoma, endometrioid adenocarcinoma and serous adenocarcinoma.	Positive CTC pre-treatment showed lack of response to mTOR inhibitor, temsirolimus and high expression of apoptosis marker in CTCs was associated with longer progression-free survival [135].
NCT00156273. 2008. Observational.	NA	Breast cancer	Advanced (Stage IV). Metastatic breast cancer. ECOG status 0–2.	In patients with elevated CTC, higher levels of CTC-apoptosis were associated with worse prognosis, while higher CTC-BCL-2 levels correlated with better outcomes [136].
NCT00967031. 2012. Interventional. Phase 2.	LANDSCAPE	Breast cancer	Advanced. Brain metastases overexpressing HER2. ECOG performance status of 0–2.	After 21 days of lapatinib treatment, a disappearance of CTC was observed in 11 of 36 patients. The 1-year overall survival rate was 83.9% in patients with no CTC at day 21 versus 42.9% in patients with ≥1 CTC [137].
NCT00428896. 2008. Interventional. Phase 2.	NA	Breast Cancer	Advanced. Metastatic breast cancer with EGFR expression.	A median reduction of 96.4 and 94.1% in CTC count was observed in 11 (64.7%) and 12 (70.6%) of patients after the first and the second gefitinib treatment cycles, respectively. Treatment-resistant CTCs could be eliminated by gefitinib in metastatic breast cancer, and EGFR expression on CTCs merits further validation as a potential biomarker for specific and effective targeting of CTCs [138].
NCT00382018. 2017. Interventional. Phase 3.	SWOG S0500	Breast Cancer	Advanced. Metastatic breast cancer. ECOG status 0–2. Patients enrolled before initiation of first line of chemotherapy. ER-positive, HER2-negative, triple-negative and HER2-positive patients were included in the study.	Prognostic significance of CTCs in patients with metastatic breast cancer receiving first-line chemotherapy was confirmed. For patients with persistently increased CTCs after 21 days of first-line chemotherapy, early switching to an alternate cytotoxic therapy was not effective in prolonging overall survival [130].
NCT01349842. 2018. Interventional. Phase 3.	CirCe01	Breast Cancer	Advanced (Stage III–IV). Metastatic lobular or ductal adenocarcinoma. Eastern Cooperative Oncology Group (ECOG) status 0–4.	Early changes in CTC count were correlated with first cycle of third line chemotherapy treatment outcome. Among patients with <5 CTC/7.5 mL at baseline showed better prognostication for progression-free survival [134]. However, due to the limited accrual and compliance, this trial failed to demonstrate the clinical utility of CTC monitoring in third- and fourth-lines chemotherapy [139].
NCT01722903. 2015. Observational.	NA	Colorectal Cancer	Advanced (Stage IV). Colorectal cancer with resectable metastases limited to liver and lungs.	CTCs were quantified in blood of patients collected at incision, during resection, 30 min after resection, and on postoperative day 1 by EpCAM-based CellSearch and size-based isolation method. CTC quantity was significantly higher with size-based filtration method than CellSearch at all points of blood collection [140].
NCT01322893. 2016. Observational.	CTC-MBC	Breast Cancer	Advanced (Stage IV). Metastatic breast cancer with estrogen receptor alpha and HER2 expression. Invasive lobular and ductal carcinoma of no special type. ECOG status 0–2.	Study demonstrated the feasibility to ascertain the status of important predictive biomarkers expressed in breast cancer CTCs using the newly developed CTC-DropMount technique [141]. Patients with a continuous presence of apoptotic or CTC clusters in follow up during systemic therapy had worse prognosis than patients without similar CTC characteristics [66]. Longitudinal evaluation of CTC and CTC clusters were shown to improve prognostication and monitoring in patients with metastatic breast cancer starting first-line systemic therapy [10]. The number of CTCs were found to be higher in invasive lobular carcinoma compared to invasive ductal carcinoma highlighting the importance of different CTC cut-off considerations in different breast cancer types [142].
NCT00694252. 2011. Interventional. Phase 2.	NA	Breast Cancer	Advanced (Stage IIIB and IV). ECOG status 0–2.	Lapatinib treatment is effective in decreasing HER2-positive CTCs in patients with metastatic breast cancer irrespective of the HER2 status of the primary tumor [143].
NCT01713699. 2017. Interventional.	NA	Leptomeningeal metastases from 9 tumor types *	Advanced. Patients treated for advanced EpCAM-positive solid tumors. ECOG status 0–4.	EpCAM-based flow cytometry assay to detect CTCs in cerebrospinal fluid is superior to cytology for the diagnosis of leptomeningeal metastases in patients with a clinical suspicion of metastases but a negative or inconclusive MRI [144].
NCT02075606. 2017. Interventional. Phase 4.	CALMNET	Neuroendocrine cancers ^#^ Midgut neuroendocrine cancers ^%^	Early and advanced. Only patients with well or moderately differentiated tumors with a Ki67 proliferation index of <20% was recruited.	Somatostatin receptors 2 and 5 were detected on CTCs in patients with neuroendocrine tumors which might be a useful biomarker for evaluating somatostatin receptor-targeted therapies [145]. Patients without CTC at baseline may be more likely to achieve a symptomatic response following lanreotide autogel treatment than patients with CTC [146].
NCT01577511. 2017. Observational.	NA	Colorectal Cancer	Advanced (Stage IV). Chemotherapy-naïve patients with metastatic colorectal cancer.	Patient-derived colorectal CTC lines contain functional cancer stem cells and express high levels of drug metabolism genes rendering them resistant to conventional therapies [147].
NCT01439568. 2016. Interventional. Phase 2.	NA	SCLC	Advanced. A total of 60–70% of patients had extensive-stage disease.	Weak positive correlation at baseline between CXCR4 expression in tumor tissue and CTCs was observed in patients treated with CXCR4 peptide antagonist LY2510924 plus carboplatin-etoposide. Baseline CXCR4+ CTCs ≥ 7% was prognostic of shorter progression-free survival [148].
NCT00898014. 2010. Observational.	IC2006-04	Breast Cancer	Advanced (Stage IV). No prior chemotherapy for metastatic disease.	Detectable CTC was the only factor observed to be significantly associated with an increased risk of arterial thrombotic events [149].
NCT01625702. 2015. Interventional.	NA	Gastric cancer	Advanced gastric adenocarcinoma. Karnofsky performance status ≥ 60.	CTC number was found to be significantly correlated to prognosis in histologically HER2-negative patients treated with fluorouracil-based chemotherapy. In patients that are histologically HER2-positive, CTC number was not obviously correlated to the progression-free or overall survival during combined anti-HER2-targeted therapy [131].
NCT02372448. 2019. Interventional.	STALKLUNG01	NSCLC	Early and advanced. Lung adenocarcinoma with ALK rearrangement on tumor tissue was included.	As a part of standardization of the pre-analytical conditions for CTC-based clinical trials, study found out that blood processed after 24 h and 48 h in BCT tubes showed stable CTCs counts and integrity, whereas CTCs in K3EDTA tubes showed an altered morphology in all patients. Moreover, CTCs recovered in BCT or K3EDTA tubes were evaluable for MET expression, ALK rearrangement studies [150]. CTCs can be used as a complementary tool to a tissue biopsy for the detection of ALK rearrangements. Longitudinal analyses of CTCs are promising for real-time patient monitoring and improved delivery of molecularly guided therapy [151].
NCT01548677. 2017. Interventional. Phase 2.	TREAT-CTC	Breast Cancer	Early. HER2-negative primary non metastatic adenocarcinoma of the breast.	Study aimed to assess whether trastuzumab treatment decreases the detection rate of CTCs in HER2 nonamplified, early breast cancer patients and found that Trastuzumab does not decrease the detection rate of CTCs [152].
NCT02937116. 2020. Interventional. Phase 1.	IBI308	Ten types of gastrointestinal tumors ^@^	Advanced (Stage IIIB-IV). ECOG status 0–1.	Abundance of PD-L1^high^ CTCs at baseline serve as a predictor to screen patients for PD-1/PD-L1 blockade therapies and measuring the dynamic changes in CTC indicate the therapeutic response at early time [153].
NCT03032913. 2017. Observational.	PANC-CTC	Pancreatic cancer	Early (Stage I, IIb and III). Pancreatic ductal adenocarcinoma.	Combined CTC and exosome detection displayed 100% of sensitivity and 80% of specificity, with a negative predictive value of 100%. High levels of exosomes and/or CTC presence were significantly correlated with progression-free survival and with overall survival when CTC clusters were found [154].
NCT01975142. 2019. Interventional. Phase 2.	CirCe T-DM1	Breast Cancer	Advanced. Metastatic breast cancer. HER2-negative primary tumor. ECOG status of 0–2.	CTC with HER2 amplification can be detected in a limited subset of HER2-negative metastatic breast cancer patients indicating the importance of clonal evolutionary changes within the tumor [155].
NCT01640444 (VISNU-2). 2018. Interventional. Phase 2. NCT01640405 (VISNU-1). 2018. Interventional. Phase 3.	VISNÚ-1/2	Colorectal Cancer	Advanced. Metastatic colorectal adenocarcinoma. ECOG status of 0–1.	Elevated baseline CTCs and RAS mutations were associated with clinicopathologic features known to be associated with poor prognosis [156]. Patients with baseline CTC ≥ 3 count had poor prognosis [157]. First-line 5-fluorouracil/leucovorin, oxaliplatin, irinotecan plus bevacizumab treatment significantly improved progression-free survival in patients with ≥3 CTCs at baseline compared to 5-fluorouracil/leucovorin, oxaliplatin plus bevacizumab doublet therapy [158].
NCT01800058. 2018. Observational.	NA	Prostate Cancer	Early (Stage II and III). Prostate adenocarcinoma. Karnofsky performance score of ≥70.	Positive CTC status at diagnosis, following neoadjuvant androgen deprivation therapy, at the end of radiotherapy, and 9 months after radiotherapy was not significantly associated with any clinical or pathologic factors and overall survival [159].
NCT02005770. 2018. Interventional. Phase 4.	NA	Breast Cancer	Early (Stage 0–III). Primary preinvasive and invasive breast cancer without metastases.	Study evaluated the association of different types of anesthesia with postoperative CTC counts in surgically resectable breast cancer patients and found that there was no difference between sevoflurane and propofol with respect to CTC counts over time [160].
NCT02453139. 2017. Interventional.	ExPeCT	Prostate Cancer	Advanced. Prostate adenocarcinoma participants were stratified based on body mass index.	Platelet cloaking of CTCs was observed in the patient population for the first time but without any significant correlation with clinico-pathological information [161].
NCT01710605. 2018. Interventional. Phase 3.	STIC CTC	Breast Cancer	Advanced. Metastatic ductal adenocarcinoma.	CTC count was found to be a reliable biomarker method for guiding the choice between chemotherapy and endocrine therapy as the first-line treatment in hormone receptor-positive, HER2-negative metastatic breast cancer patients [162].
NCT01596790. 2019. Interventional.	NA	Colorectal Cancer	Advanced. Colon or rectum adenocarcinoma, visceral metastases. WHO performance status 0, 1 or 2.	Differential gene expression pattern was observed in CTCs of same patient during first- and second-line chemotherapy treatments and disease progression highlighting the CTCs adaptability to escape treatment pressure [132].
NCT02549430. 2017. Interventional. Phase 2.	TREnd	Breast Cancer	Advanced. Endocrine resistant ER-positive, HER2-negative advanced breast adenocarcinoma.	CTC count was found to be a promising modality in monitoring palbociclib response in patients with ER-positive, HER2-negative advanced breast cancer [163].
NCT02137837. 2019. Interventional. Phase 3.	SWOG1222	Breast Cancer	Advanced. Invasive breast carcinoma with ER-positive and HER-2-negative status.	An association was observed of baseline CTC and ctDNA with poorer survival [164].
NCT02771314. 2020. Interventional. Phase 2.	NA	NSCLC	Early and advanced. Patients with histologically documented EGFR-mutant NSCLC.	The decrease in both CTCs and ctDNA occurring early during osimertinib treatment in EGFR Mutant NSCLC patients was found to be predictive of better outcome [165].
NCT03033927. 2024 (estimated). Observational.	NA	Pancreatic cancer	Advanced pancreatic adenocarcinoma.	Chemosensitivity assay profiling of CTCs was found to be a promising tool for guiding therapy in advanced pancreatic adenocarcinoma [133].
NCT03935802. 2018. Observational.	NA	Breast Cancer	Early (Stage I–III). Invasive ductal carcinoma, Invasive lobular carcinoma.	Increase in CTC numbers over the course of adjuvant radiotherapy signified a potential predictive biomarker to judge relative risk or benefit in patients with early breast cancer [166].
NCT04358718. 2021. Interventional.	NA	Bladder cancer	Early	μ-opioid receptor agonists used for pain treatment both during and after surgery in blader cancer patients was associated with high CTCs and CTC cluster counts [167].
NCT01740804. 2026. Observational.	POLICE	NSCLC	Advanced (Stage IIIb and IV). Adenocarcinoma, squamous cell carcinoma and Mixed NSCLC ECOG status of 0–1.	CTC persistent presence during treatment represented poor prognosis and resistance to chemotherapy in advanced NSCLC [129].
NCT01619111. 2022. Interventional. Phase 3.	DETECT III	Breast Cancer	Advanced. HER2+ metastatic breast cancer. ECOG Score < 2.	Study demonstrated that phenotyping of CTCs has clinical utility for stratification of metastatic breast cancer patients irrespective of HER-2-positive or -negative status for targeted therapy. Study highlighted the phenotypic changes in tumor cells during disease progression [168].

Abbreviations: NA—Not available, NSCLC—non-small-cell lung cancer, SCLC—small-cell lung cancer. * Nine tumor types including breast cancer, NSCLC, SCLC, gastrointestinal cancer, ovarian cancer, nasopharyngeal carcinoma, urothelial cell carcinoma, renal cell cancer and parotid gland carcinoma. ^#^ Study recruited patients with metastatic neuroendocrine tumors of either midgut or pancreas origin. ^@^ Ten types of gastrointestinal tumors including neuroendocrine tumors of the right adrenal neuroblastoma, hepatocellular carcinoma, colorectal carcinoma, intrahepatic cholangiocarcinoma, pancreatic carcinoma, esophageal carcinoma, ampullary adenocarcinoma, small intestinal stromal tumor, and esophageal small-cell carcinoma. **^%^** Patients with neuroendocrine tumors of the ileum, caecum, jejunum, small bowel, duodenum, and right colon were recruited in the study.
